# The Liabilities for the Treatment of Pay Patients in Hospitals

**Published:** 1899-08-26

**Authors:** 


### THE LIABILITIES FOR THE TREATMENT OF PAY PATIENTS IN HOSPITALS.

The following judgment, delivered by the Supreme Court in New York, on the liability of hospital authorities for malpractices on the part of their staff, will be of interest to many, as, although the decisions of American Courts are by no means binding in English law, they are often taken into consideration by English judges. The point of special interest is the fact that all previous decisions regarding the non-liability of hospitals for similar damages are now reversed. Justices Van Brunt, Barrett, Patterson, and Rumsey all concurred in this decision formally rendered by Justice Barrett. The following is the text of the ruling: "Though the defendant is called a charity hospital, it has its pay side, on which side it is in the habit of furnishing private rooms and nurses to well-to-do persons for a full price, thus entering into an express contract. For the breach of that contract the plaintiff is entitled to the same damages as though the action had been for negligence pure and simple, and she is entitled to an adequate compensation for her injuries. The person furnished (Miss Kinney) was not a trained nurse, but a pupil in the defendant's training school, having studied only nine months of the two years required for the course. It stands to reason that such a misfortune as happened to Miss Ward would have been less likely to occur had she been in the hands of a fully-trained nurse. It is for the jury to say whether, in furnishing this careless pupil, of limited experience, the defendant fulfilled its contract obligation to the plaintiff; and if it did not, and injury resulted from the breach of the contract, to award the adequate compensation for such injury."

The New York *Medical Record*, commenting upon the decision, goes on to say: "While this ruling is eminently just under the circumstances, it must be clearly understood that it refers to pay patients only, thus absolving from similar penalties the institutions in which patients are treated free. In the former case it is a contract for services for which money is charged, and in the other the moral obligation is involved to do the best for the sufferer on strict Samaritan principles only. Not that there is any difference in the essential duties to be performed, but in the accountability for their performance on a strictly pecuniary basis. Whenever there is a question of money in a transaction the damages are necessarily decided by a pecuniary standard. The exact contrary of this principle applies to the strictly charitable institutions. When gratuitous service is rendered, the recipient, who is the only person benefited thereby, must take them at his own risk. While wilful neglect on the part of beneficiaries is always subject to punishment, the presumption of fair dealing should always weigh in favour of the defendant, who has no real motive for wrongdoing. Pure charity should always be above such suspicion. It simply accepts results without bargaining to control them. Everything is given, and nothing is exacted but the due appreciation of kind offices rendered. In thus defining the respective rights of the pay and free patient, the line of responsibility in case of malpractice and neglect is very satisfactorily drawn. It is eminently proper that this should be the case, in view of the important issues at stake with all hospital surgeons and physicians. Everyone knows how many legal fights have been necessary before this desirable point has been reached. In future cases the latest ruling will effectually prevent any confusion of ideas as to responsibility, and will enable all free hospitals to make a good defence against the frequent and annoying suits brought by designing sharpers, arrant frauds, and shystering lawyers."

